# Variation in diagnostic test requests and outcomes: a preliminary metric for OpenPathology.net

**DOI:** 10.1038/s41598-018-23263-z

**Published:** 2018-03-19

**Authors:** Jack W. O’Sullivan, Carl Heneghan, Rafael Perera, Jason Oke, Jeffrey K. Aronson, Brian Shine, Ben Goldacre

**Affiliations:** 1 Centre for Evidence-Based Medicine, Nuffield Department of Primary Care Health Sciences, University of Oxford, Oxford, UK; 2 Department of Clinical Biochemistry, John Radcliffe Hospital, University of Oxford, Oxford, UK

## Abstract

Efforts to reduce healthcare costs have led to the development of metrics to identify unwarranted variation in care. Previous work assessing diagnostic tests is limited, despite their substantial contribution to expenditure. We explored C-reactive Protein (CRP) and Erythrocyte Sedimentation Rate (ESR) tests ordered across Oxfordshire NHS General Practices, and the proportion of tests that yielded an abnormal result, and identified practices that had a proportion of abnormal CRP and ESR results 3 standard deviations below the mean. We estimated the adjusted average proportion of abnormal CRP and ESR tests that yielded abnormal results from each practice, after adjusting for differences in practice populations. These proportions were plotted against the total CRP and ESR requests per practice. We constructed funnel plots to identify practices 3 standard deviations below the mean proportion of abnormal CRP and ESR tests. We analysed 143,745 CRP and 30,758 ESR requests from 69 practices. Twelve (17%) and 7 (10%) practices were more than 3 standard deviations below the mean for CRP and ESR testing respectively. Two practices (3%) were below the 99.8% limit for both CRP and ESR ordering. Variation in the proportion of tests with an abnormal result shows promise for auditing variation in care.

## Introduction

Unwarranted variation in the use of healthcare resources contributes substantially to excessive expenditure^1–5^; currently around 25% of healthcare spend globally is estimated to be inappropriate ‘overuse’^6,7^. Various metrics have been developed to audit inappropriate care, and a 2012 systematic review shows that data feedback to practitioners can have a modest positive impact on future behaviour^8^. However, although laboratory tests cost the NHS around £2.5 billion annually^9^ and influences more than 70% of clinical decisions^9^, there are few measures examining diagnostic test ordering. Metrics are currently limited to examining geographical variation in the crude volume of test requests^10,11^, and we are aware of no work using data feedback to monitor or modify behaviour.

Primary care represents the ideal setting to mitigate unwarranted diagnostic test use. 90% of UK consultations occur in General Practice^12^, and tests are requested commonly: more than one-third of all laboratory tests in the UK are requested from primary care^9^.

There is evidence to suggest that the ordering of C-reactive Protein (CRP) and Erythrocyte Sedimentation Rate (ESR) tests from General Practice can be improved. UK General Practitioners (GPs) feel uncertain about the appropriate use of inflammatory marker tests (CRP, ESR) and ‘differ in their approach’ to ordering^13^. Utilisation has also increased substantially – of all laboratory tests, CRP and ESR had the second and 15^th^ largest increase in requests between 2005 and 2009^14^, and there is substantial geographical variation in the crude rate of requesting^14^. Furthermore, inflammatory markers are used diagnostically rather than for monitoring. Therefore, variation in usage reflects a different approach to the use of a blood test in suspected new diagnoses, rather than variation in the frequency of routine monitoring of disease progression.

We therefore set out to develop a practice level assessment of CRP and ESR requests from Oxfordshire General Practices. Specifically, we set out to calculate the number of requests, and the annual proportion of CRP and ESR tests that returned an abnormal result for each practice. We subsequently identified practices with a proportion of abnormal CRP and ESR tests that was 3 standard deviations below the mean of their colleagues.

## Methods

We conducted this study in line with STROBE (Strengthening the Reporting of Observational Studies in Epidemiology)^15^ and RECORD (REporting of studies Conducted using Observational Routinely-collected health Data)^16^ Statements.

### Study design and setting

We retrospectively analysed all CRP and ESR tests requested from Oxfordshire General Practices from 1^st^ January 2016 to 31^st^ December 2016, using data from the clinical laboratories of the Oxford University Hospitals (OUH) Trust. Practices that send most of their laboratory requests to other laboratories or without practice demographic data were excluded.

### Data source

This study was conducted as a service evaluation using previously collected, non-identifiable data. The OUH Research and Development Department approved access to the data (study identifier: CSS-BIO-4-4935). We collected routine electronic CRP and ESR test results held by the OUH laboratory, which included the date of the test, anonymised patient ID, the result, and the unique National General Practice Code. We used the National General Practice codes to identify each Practice’s 2016 list size, age and gender breakdown and their Indices of Multiple Deprivation (IMD) via Public Health England^17^ and NHS Business Authority (data corresponding to practice demographics relate to Q2 of 2016 (April to June)^18^.

The original data file contained quantitative data on the CRP and ESR test results in mg/L and mm/h respectively. We dichotomised CRP results into ‘abnormal’ (>5 mg/L) or ‘normal’ (<5 mg/L), and ESR results into ‘abnormal’ (>20 mm/h for female patients, >14 mm/h for male patients) or normal (<20  and <14 mm/h respectively) on the advice of a Consultant Chemical Pathologist and custodian of OUH laboratory information (BS) in accordance with the immunoturbidimetric assay used at OUH laboratories (Abbot Architect chemistry analyser (Abbott Diagnostics, Maidenhead, UK)). We calculated the proportion of abnormal CRP and ESR tests ordered for each practice, as “the number of abnormal CRP tests”/“number of CRP tests ordered” and “the number of abnormal ESR tests”/“number of ESR tests ordered”).

### Statistical analysis

We constructed two separate statistical models exploring the variation in the proportion of abnormal CRP and ESR tests across practices using methods adopted from Spiegelhalter’s method for handling over-dispersion of performance indicators^19^. Spiegelhalter suggests using a random-effects model to compare institutional performance^19^, as it controls for ‘unmeasured factors that lead to systematic differences between the true underlying rates in institutions’^19^. We therefore used a generalised linear mixed-effects regression model with binomial link function to estimate an ‘adjusted average proportion abnormal’ of CRP tests and ESR tests for each General Practice. We adjusted for differences in practice populations between practices; namely differences in age, gender and deprivation. We also adjusted for the difference in the proportion of CRP and ESR testing that was ‘repeat’ testing – tests ordered for patients that received >1 CRP or ESR test in 2016. We adjusted for differences in age and gender by calculating the proportion of a General Practice population >65 years and female. The 2016 practice-specific IMD scores were used to adjust for differences in deprivation. We also used a random effects model to account for other unmeasured differences between practices. These model-predicted adjusted proportions of abnormal CRP and ESR tests for each practice represent practice-level estimates, accounting for any differences in age, gender, repeat testing and deprivation and other unmeasured differences between practices. Practice list size was ultimately dropped from the model due to co-linearity with the total number of CRP and ESR tests ordered per practice, which was included in the model. Gender was similarly dropped, as it did not improve fit.

We constructed funnel plots to display the variation in the proportion of abnormal CRP and ESR tests between practices. Funnel plots show an ‘observed indicator against a measure of its precision, typically the sample size’^19^. We plotted 95% and 99.8% control limits corresponding to two and three standard deviations (SD) from the mean^19^. We also plotted the crude and adjusted proportion of abnormal CRP and ESR tests requested for each General Practice against the total number of CRP and ESR tests ordered per practice. All analyses were performed in R (version 3.4.1).

### Data availability

The unadjusted average proportion of abnormal CRP and ESR tests and total tests ordered per practice analysed during the current study are available in the Supplementary file. The data file is available upon reasonable request and the code used to produce the results is published on GitHub (https://github.com/jackosullivanoxford/Scientific-Reports-Paper/blob/master/R_code).

## Results

### Descriptive data

We analysed 143,745 and 30,758 CRP and ESR tests from 69 General Practices over 2016; on average each practice ordered 2083 CRP and 446 ESR tests annually (Table 1).

**Table 1:** Practice Characteristics.

Practice Characteristics	Number
Number of General Practices	69
Total number of CRP tests ordered	143,745
Total number of ESR tests ordered	30,758
Median Practice List Size (IQR: Q1 to Q3)	8243 (5342 to 11,509)
Median IMD Decile (IQR: Q1 to Q3)	9 (7 to 10)
Mean number of CRP tests ordered per practice annually (SD)	2083 (1044)
Mean number of ESR tests ordered per practice annually (SD)	446 (400)

### Variation in the crude proportion of abnormal CRP and ESR tests ordered

**Figure 1:**
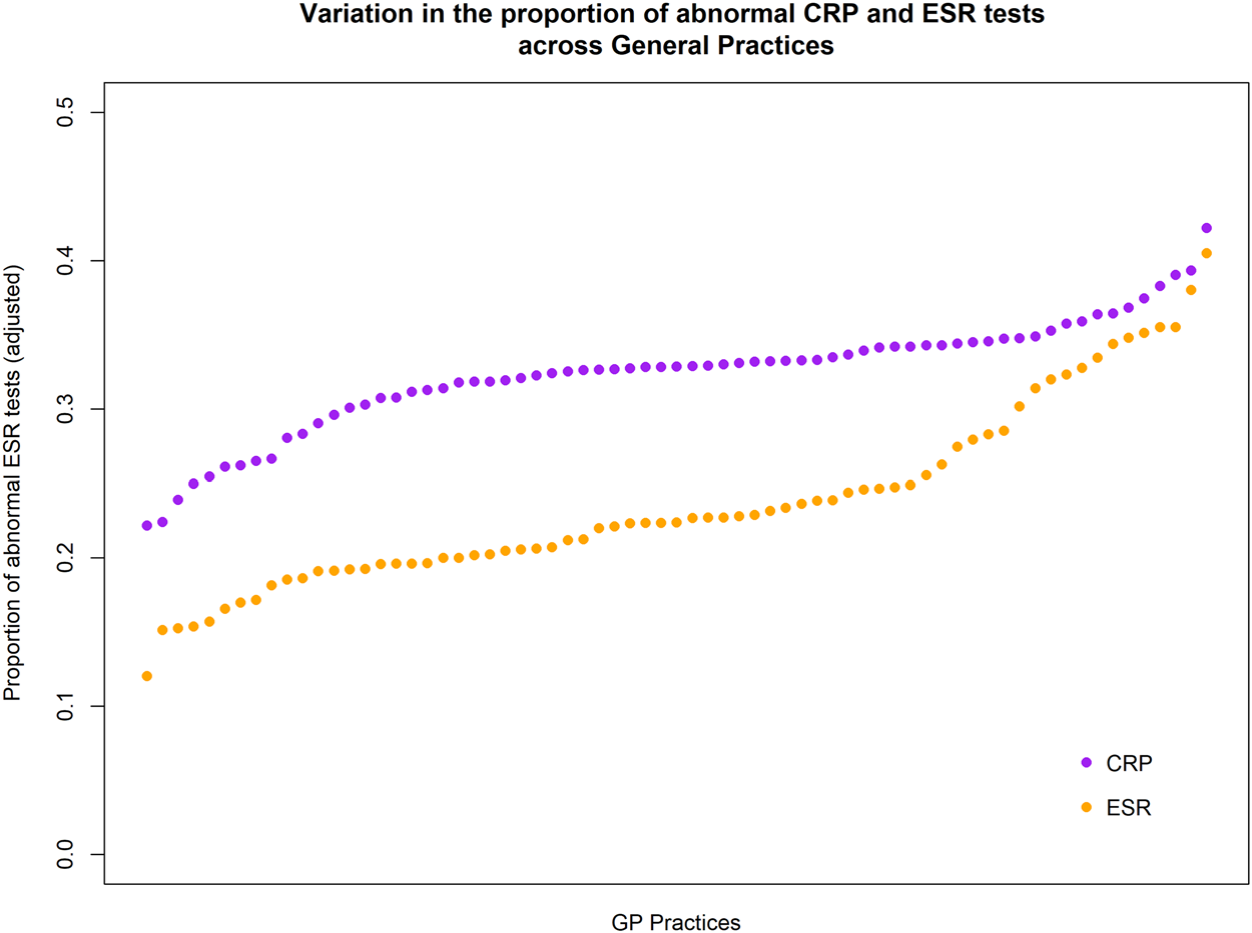
Variation in the proportion of abnormal CRP and ESR tests across Oxfordshire General Practices.

Figure 1 shows the variation in the adjusted proportion of abnormal CRP and ESR tests requested for each General Practice. The annual proportion of CRP tests that returned an abnormal result varied from 22% to 42%, whereas the proportion of abnormal ESR tests varied substantially more, from 12% to 41%.

### Random effects binomial regression model

**Figure 2:**
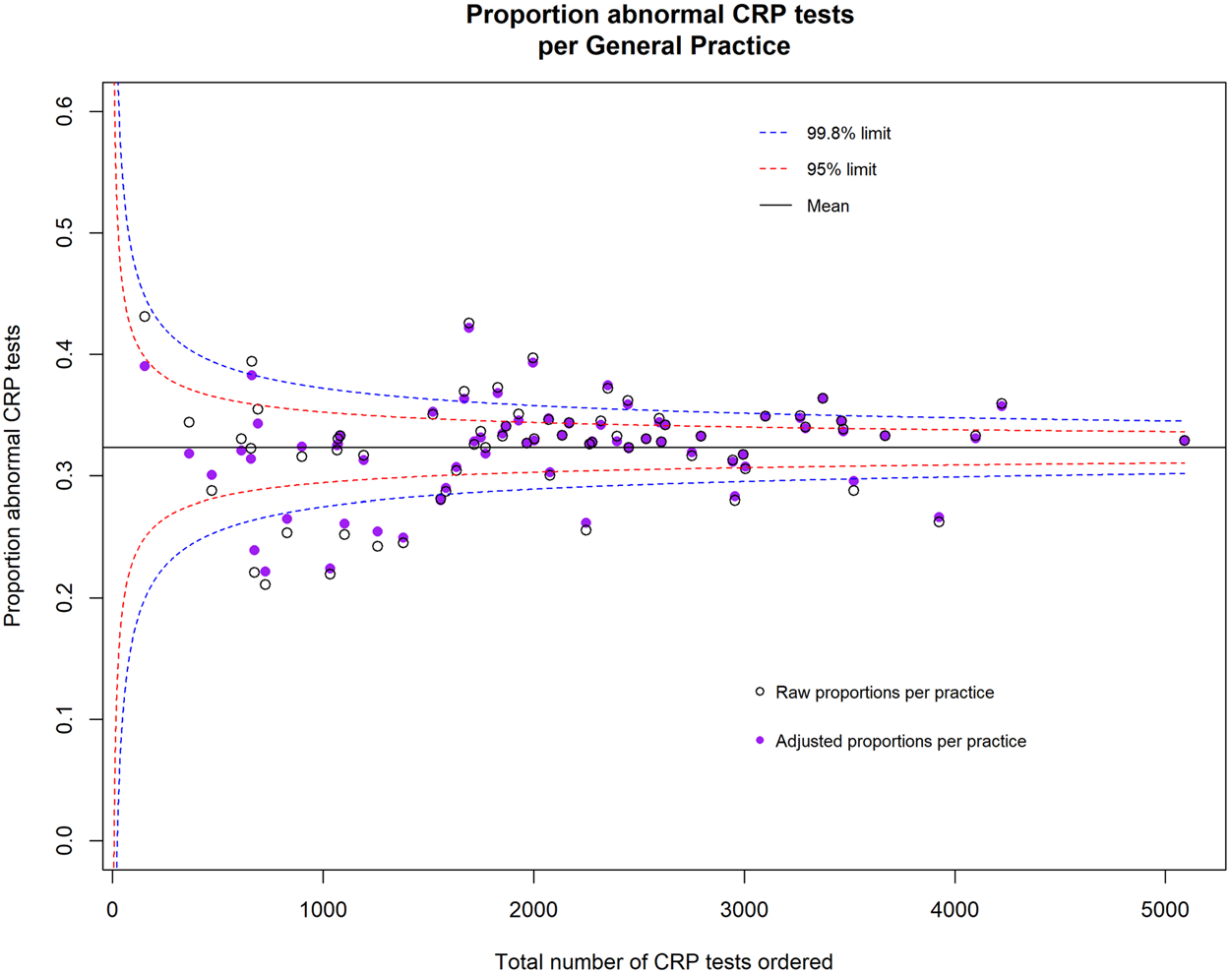
Funnel plot comparison of adjusted and unadjusted (raw) average proportion of abnormal CRP tests per GP practice.

**Figure 3:**
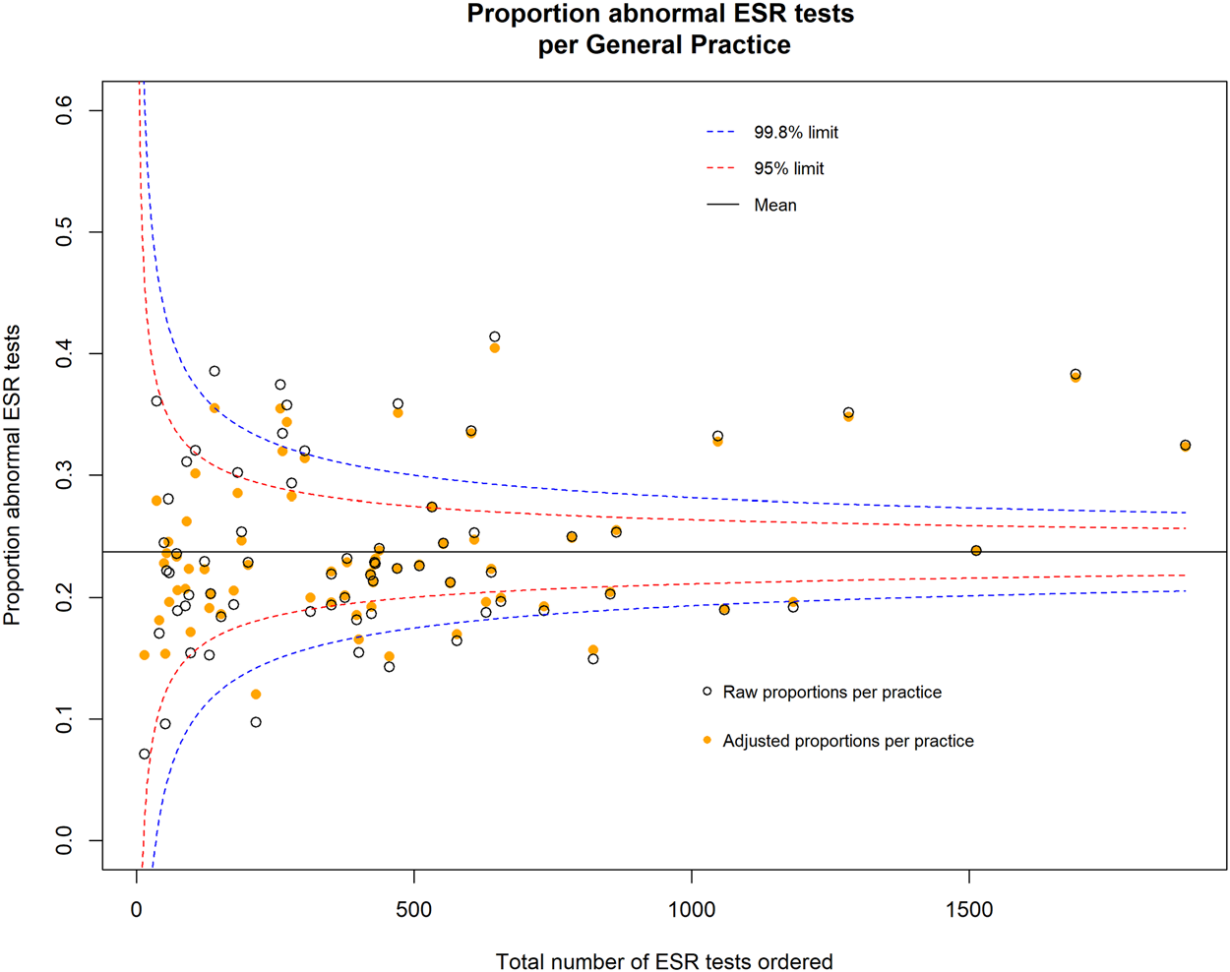
Funnel plot comparison of adjusted and unadjusted (raw) average proportion of abnormal ESR tests per GP practice.

We report the raw (unadjusted) and adjusted proportions of abnormal CRP and ESR tests for each practice (Supplementary File). Figures 2 and 3 show the effect of the binomial regression model on the proportions of abnormal CRP and ESR tests for each practice. Table 2 shows the effect of the model on number of practices within the 95% and 99.8% control limits. As expected, the model brings each General Practice’s proportion of abnormal CRP and ESR testing towards the mean, and this effect diminishes with increasing sample size.

**Table 2:** Comparison of adjusted and unadjusted models.

Model	Number of practices within 2 SD of mean (%)	Number of practices beyond 2 SD of mean (above or below) but within 3 SDs (%)	Number of practices beyond 3 SD of mean; above or below (%)	Number of practices *below* 3 SDs from mean
CRP Adjusted	38 (55%)	11 (16%)	20 (29%)	12 (17%)
CRP Unadjusted	33 (48%)	15 (22%)	21 (30%)	12 (17%)
ESR Adjusted	43 (62%)	10 (15%)	16 (23%)	7 (10%)
ESR Unadjusted	37 (54%)	13 (19%)	19 (28%)	7 (10%)

The mean proportions of abnormal CRP and ESR tests among all Oxfordshire General Practices were 0.32 and 0.24 respectively. For both the CRP and ESR model, most of the practices (CRP: n = 38; 55%, ESR: n = 43, 62%) were within 2 standard deviations of the mean (Table 2). Regarding the CRP model, the proportions of abnormal CRP tests ordered for 20 practices (29%) were greater than 3 standard deviations from the mean, of which 12 practices (17%) were *below* the 99.8% limit (3 standard deviations). Eleven practices (16%) had proportions greater than 2 but less than 3 standard deviations from the mean, two of these practices (2.9%) were *below* the 95% limit (2 standard deviations) (Table 2).

The proportions of abnormal ESR tests ordered for 16 practices (23%) were greater than 3 standard deviations from the mean, of which 7 practices (10%) were *below* the 99.8% limit. Ten practices (15%) had proportions greater than 2 but less than 3 standard deviations from the mean, six of these practices (6.7%) were *below* the 95% limit (Table 2).

Two practices (3%) were below the 99.8% control limits for both CRP and ESR ordering, while three practices (4%) were above the 99.8% limit for both CRP and ESR ordering.

## Discussion

### Summary of findings

Data to generate a potential new metric for audit and feedback of inflammatory marker test usage in primary care were successfully acquired and processed. After adjustment, 12 (17%) and seven (10%) practices had a proportion of abnormal CRP and ESR tests more than 3 standard deviations below the mean. Two practices (3%) were below the 99.8% control limit for both CRP and ESR ordering. Adjustment consisted of controlling for differences in age, gender, deprivation and the total and proportion of repeat CRP and ESR tests between general practices, as well as other unknown differences between the practices in the random effects model.

### Comparison with previous literature

To our knowledge there is no previous work describing the variation in the proportion of abnormal CRP or ESR tests between General Practices, or indeed variation in the proportion of abnormal results for any other test. However, the UK’s National Audit Office has developed a similar process for generating the proportion of ‘positive’ referrals to the 2-Week Wait (2WW) cancer pathway^20^. They monitor and make available the ‘conversion rate’ of referrals GPs make to specialists under the 2WW pathway^21^. The results are stratified by Clinical Commissioning Group (CCG) and can be compared online^21^.

The use of CRP and ESR in UK General Practice has been examined quantitatively^14^ and qualitatively^13^. From 2005 to 2009, CRP use rose 86%, second only to faecal occult blood^14^, ESR use also rose, by 24%. Similarly, requests for CRP and ESR had substantial geographical variation (ESR and CRP were the 11^th^ and 14^th^ most variable lab tests)^14^. Qualitative work has shown that UK GPs feel uncertain about when to order CRP and ESR tests and that they differ in how they incorporate the use of these tests into their approach to patients with undifferentiated symptoms^13^.

### Strengths and limitations

We have applied robust methods to adjust for known and unknown factors that may account for variations across GP practices, applying an appropriately conservative random-effects model. While it is desirable to account for unknown differences between practices our approach may be excessively conservative, obfuscating GP practices that are truly 2 or 3 standard deviations divergent from the mean^19^. We were unable to identify any further data sources for explanatory variables in our model, such as disease prevalence by small local area, and could therefore only account for differences in age, gender, deprivation, repeat testing and the number of CRP tests requested. There may therefore be other unmeasured and unmeasurable confounders.

A further limitation is the generalisability of our data. We only included data from Oxfordshire General Practices, and thus our results do not reflect UK primary care nationally and may not be consistent across the UK. Our methods, however, are still applicable in other regions and at a national level. Furthermore, inferences about individual patients from our results would be inappropriate and represent an ecological fallacy. We only suggest our results to be used to identify general practices that would benefit from further investigation; the practice is the unit of analysis, and the unit of interest, not the patient.

Examining CRP and ESR is a strength for many reasons. There is prior evidence that UK GPs are uncertain when and how to use CRP and ESR tests^13^, and there is temporal and geographical variation in the number of CRP and ESR test requests in the UK^14^. Unlike radiological or other diagnostic services, CRP and ESR results can be clearly dichotomised into normal and abnormal.

### Clinical implications

Ultimately, we envisage that data comparing the proportion of abnormal test results could be used as a feedback tool to give GPs information about their use of tests or for monitoring of GPs at a CCG or national level. Similar feedback is already given to GPs regarding, for example, antibiotic prescribing, with some evidence of impact on prescribing^22,23^. As with all audit and data feedback, such information is best thought of as a *measure* rather than an *indicator*, requiring judicious interpretation in the context of population and patient data^24,25^. In this regard, we do not want our data to imply that GPs three standard deviations above or below the mean of their colleagues are ordering CRP and ESR tests inappropriately. Appropriateness of test ordering should be judged using patient level data; our data can only determine GPs that would benefit from patient-level audit. For instance, as pressure to reduce waste continues to increase in the NHS, the Oxfordshire CCG could direct resources to auditing the CRP ordering of the 12 practices with an average proportion of abnormal results below 3 standard deviations, rather than all 69 practices. Our methods can be applied to other diagnostic tests and at a national level as an initial step in trying to identify inappropriate use of diagnostic tests.

We suggest that practices *below* (rather than above) the lower 99.8% control limits should be the focus of audit, particularly practices that order many CRP or ESR tests, as this may reflect excessive or inappropriate use in patients with a low pre-test probability of an abnormal result. Interpretation of practices with a proportion of abnormal tests above the upper 99.8% control limit is more complex. This could represent GPs with astute history and physical examination skills confirming a diagnosis; or GPs with an excessively high threshold for ordering a CRP or ESR; or GPs that ordered CRP or ESR tests more commonly for patients with a high pre-test probability, for instance using CRP or ESR to monitor chronic disease. Some of our team are involved in delivering OpenPrescribing.net: an openly accessible, open source, and open data tool that provides easy access to various analytic approaches to identify outliers for prescribing behaviour in primary care. With new access to data we are now building the specifications for OpenPathology, taking a similar approach to pathology metrics.

### Future Research

Future research should focus on assessment of the appropriateness of CRP and ESR requests among a range of abnormal and normal CRP and ESR requesters, using individual patient data (IPD) either in the form of notes review, GP interview or, if possible, IPD data audit. An assessment of the effect of our data as a GP feedback tool would also be of value, ideally in the context of a randomised controlled trial examining the impact of data feedback on the proportion of abnormal tests ordered.

## Conclusion

We used robust and conservative methods to identify two practices (3%) that requested a significantly lower proportion of CRP and ESR tests yielding abnormal results. We also identified 12 (17%) and seven (10%) General Practices that requested a significantly lower proportion of CRP and ESR tests yielding abnormal results, respectively. Variation in the proportion of tests with an abnormal result shows promise as an automated tool for auditing variation in care, and may contribute to improving quality and cost effectiveness.

## Electronic supplementary material

Supplementary file

Strobe Checklist

Record Checklist
