# Author Correction: Variation in diagnostic test requests and outcomes: a preliminary metric for OpenPathology.net

**DOI:** 10.1038/s41598-018-26376-7

**Published:** 2018-05-24

**Authors:** Jack W. O’Sullivan, Carl Heneghan, Rafael Perera, Jason Oke, Jeffrey K. Aronson, Brian Shine, Ben Goldacre

**Affiliations:** 1 Centre for Evidence-Based Medicine, Nuffield Department of Primary Care Health Sciences, University of Oxford, Oxford, UK; 2 Department of Clinical Biochemistry, John Radcliffe Hospital, University of Oxford, Oxford, UK

Correction to: *Scientific Reports* 10.1038/s41598-018-23263-z, published online 19 March 2018

This Article contains errors in Reference 25, which is incorrectly given as:

‘O’Sullivan, J. W*. et al*. Overtesting and undertesting in primary care: a systematic review and meta-analysis BMJ Open 2018;8:e018557. 10.1136/bmjopen-2017-0185578557.’

The correct reference is listed below as ref. 1.

## References

[1] O’Sullivan JW (2018). Overtesting and undertesting in primary care: a systematic review and meta-analysis. BMJ Open.

