# Supplementary material for: Variation in diagnostic test requests and outcomes: a preliminary metric for OpenPathology.net
Source: Sci Rep. 2018 Mar 19;8:4752. doi: 10.1038/s41598-018-23263-z (PMC5859290; doi:10.1038/s41598-018-23263-z)
Supplement: Supplementary file 1 — Supplementary file [file 41598_2018_23263_MOESM1_ESM.docx]

**Variation in diagnostic test requests and outcomes: a preliminary metric for OpenPathology.net**

O’Sullivan JW^1*^, Heneghan C^1^, Perera R^1^, Oke J^1^, Aronson J^1^, Shine B^2^, Goldacre B^1^.

^1^ Centre for Evidence-Based Medicine, Nuffield Department of Primary Care Health Sciences, University of Oxford, UK

^2^Department of Clinical Biochemistry, John Radcliffe Hospital, University of Oxford, UK

Jack W O’Sullivan, Clinical Researcher, [jack.osullivan@phc.ox.ac.uk](mailto:jack.osullivan@phc.ox.ac.uk)

Carl Heneghan, Professor of Evidence-Based Medicine, [carl.heneghan@phc.ox.ac.uk](mailto:carl.heneghan@phc.ox.ac.uk)

Rafael Perera, Professor of Medical Statistics, [rafael.perera@phc.ox.ac.uk](mailto:rafael.perera@phc.ox.ac.uk)

Jason Oke, Senior Statistician, [jason.oke@phc.ox.ac.uk](mailto:jason.oke@phc.ox.ac.uk)

Jeffrey Aronson, Reader in Evidence-Based Medicine, [jeffrey.aronson@phc.ox.ac.uk](mailto:jeffrey.aronson@phc.ox.ac.uk)

Brain Shine, Consultant Chemical Pathologist, [Brian.Shine@ouh.nhs.uk](mailto:Brian.Shine@ouh.nhs.uk)

Ben Goldacre, Senior Clinical Research Fellow, [ben.goldacre@phc.ox.ac.uk](mailto:ben.goldacre@phc.ox.ac.uk)

**Supplementary File**

| Practice ID | Adjusted average proportion of abnormal ESR tests | Unadjusted average proportion of abnormal ESR tests | Total ESR tests ordered | Adjusted average proportion of abnormal CRP tests | Unadjusted average proportion of abnormal CRP tests | Total CRP tests ordered |
| --- | --- | --- | --- | --- | --- | --- |
| 1 | 0.249098 | 0.25 | 784 | 0.262037 | 0.255783 | 2248 |
| 2 | 0.223219 | 0.229508 | 122 | 0.363702 | 0.369461 | 1670 |
| 3 | 0.153799 | 0.096154 | 52 | 0.261058 | 0.252269 | 1102 |
| 4 | 0.165622 | 0.155 | 400 | 0.249819 | 0.245474 | 1381 |
| 5 | 0.19622 | 0.187599 | 629 | 0.254637 | 0.242448 | 1258 |
| 6 | 0.181387 | 0.170732 | 41 | 0.224107 | 0.219536 | 1034 |
| 7 | 0.223473 | 0.202128 | 94 | 0.314022 | 0.322679 | 657 |
| 8 | 0.18626 | 0.184211 | 152 | 0.322787 | 0.323409 | 2452 |
| 9 | 0.191368 | 0.152672 | 131 | 0.318597 | 0.344353 | 363 |
| 10 | 0.202515 | 0.203008 | 133 | 0.326114 | 0.326269 | 2265 |
| 11 | 0.228991 | 0.23219 | 379 | 0.374675 | 0.372024 | 2352 |
| 12 | 0.207168 | 0.193182 | 88 | 0.332636 | 0.333025 | 1081 |
| 13 | 0.236322 | 0.222222 | 54 | 0.422029 | 0.425871 | 1693 |
| 14 | 0.120509 | 0.097674 | 215 | 0.331811 | 0.336764 | 1749 |
| 15 | 0.200024 | 0.196646 | 656 | 0.347439 | 0.34621 | 2071 |
| 16 | 0.227228 | 0.225933 | 509 | 0.33269 | 0.333061 | 3669 |
| 17 | 0.192391 | 0.186761 | 423 | 0.319374 | 0.316727 | 2750 |
| 18 | 0.185493 | 0.181818 | 396 | 0.325242 | 0.321462 | 1067 |
| 19 | 0.231789 | 0.227907 | 430 | 0.326767 | 0.326894 | 1967 |
| 20 | 0.24669 | 0.253968 | 189 | 0.307597 | 0.305925 | 3004 |
| 21 | 0.206154 | 0.189189 | 74 | 0.343057 | 0.355072 | 690 |
| 22 | 0.22705 | 0.228972 | 428 | 0.334889 | 0.332793 | 1851 |
| 23 | 0.195901 | 0.193732 | 351 | 0.382956 | 0.39426 | 662 |
| 24 | 0.190941 | 0.189802 | 1059 | 0.33091 | 0.333008 | 4099 |
| 25 | 0.274649 | 0.274436 | 532 | 0.345126 | 0.345276 | 3461 |
| 26 | 0.255595 | 0.253472 | 864 | 0.329283 | 0.330504 | 2003 |
| 27 | 0.27942 | 0.361111 | 36 | 0.320793 | 0.330606 | 611 |
| 28 | 0.151506 | 0.142857 | 455 | 0.283214 | 0.280108 | 2956 |
| 29 | 0.314102 | 0.320132 | 303 | 0.341384 | 0.341006 | 1868 |
| 30 | 0.20582 | 0.194286 | 175 | 0.307482 | 0.304534 | 1632 |
| 31 | 0.171587 | 0.154639 | 97 | 0.302994 | 0.300578 | 2076 |
| 32 | 0.245864 | 0.280702 | 57 | 0.22189 | 0.211034 | 725 |
| 33 | 0.22394 | 0.223881 | 469 | 0.33931 | 0.340122 | 3290 |
| 34 | 0.247565 | 0.253289 | 608 | 0.311518 | 0.313179 | 2944 |
| 35 | 0.19641 | 0.220339 | 59 | 0.280564 | 0.28113 | 1558 |
| 36 | 0.169774 | 0.164645 | 577 | 0.349038 | 0.349145 | 3099 |
| 37 | 0.15263 | 0.071429 | 14 | 0.390376 | 0.431373 | 153 |
| 38 | 0.238568 | 0.238757 | 1512 | 0.328851 | 0.329273 | 5090 |
| 39 | 0.223604 | 0.220657 | 639 | 0.328627 | 0.332777 | 2395 |
| 40 | 0.238758 | 0.240275 | 437 | 0.332329 | 0.332856 | 2794 |
| 41 | 0.243861 | 0.244565 | 552 | 0.327481 | 0.328089 | 2606 |
| 42 | 0.204713 | 0.202814 | 853 | 0.345554 | 0.350959 | 1929 |
| 43 | 0.211888 | 0.212389 | 565 | 0.344276 | 0.347458 | 2596 |
| 44 | 0.226802 | 0.228856 | 201 | 0.324132 | 0.315907 | 899 |
| 45 | 0.201822 | 0.2 | 375 | 0.290282 | 0.287429 | 1583 |
| 46 | 0.199989 | 0.188498 | 313 | 0.328451 | 0.32596 | 1718 |
| 47 | 0.157064 | 0.149635 | 822 | 0.318591 | 0.323546 | 1771 |
| 48 | 0.19624 | 0.191885 | 1183 | 0.336693 | 0.338617 | 3470 |
| 49 | 0.192618 | 0.189373 | 734 | 0.347636 | 0.349663 | 3266 |
| 50 | 0.21993 | 0.218527 | 421 | 0.328321 | 0.327919 | 2278 |
| 51 | 0.233643 | 0.236111 | 72 | 0.342967 | 0.343793 | 2167 |
| 52 | 0.228171 | 0.244898 | 49 | 0.368238 | 0.372881 | 1829 |
| 53 | 0.283076 | 0.293907 | 279 | 0.265054 | 0.253623 | 828 |
| 54 | 0.320004 | 0.334601 | 263 | 0.352685 | 0.350658 | 1520 |
| 55 | 0.334528 | 0.33665 | 603 | 0.312891 | 0.316848 | 1193 |
| 56 | 0.380329 | 0.383205 | 1691 | 0.330093 | 0.330702 | 2534 |
| 57 | 0.355271 | 0.385714 | 140 | 0.295993 | 0.287866 | 3519 |
| 58 | 0.355076 | 0.374517 | 259 | 0.333127 | 0.333646 | 2134 |
| 59 | 0.262564 | 0.311111 | 90 | 0.341991 | 0.345125 | 2318 |
| 60 | 0.327801 | 0.332378 | 1047 | 0.357451 | 0.359545 | 4222 |
| 61 | 0.212604 | 0.213615 | 426 | 0.31787 | 0.317635 | 2994 |
| 62 | 0.343735 | 0.357934 | 271 | 0.239154 | 0.221068 | 674 |
| 63 | 0.301674 | 0.320755 | 106 | 0.300869 | 0.288136 | 472 |
| 64 | 0.323381 | 0.32504 | 1889 | 0.266461 | 0.262742 | 3924 |
| 65 | 0.348004 | 0.351794 | 1282 | 0.342103 | 0.342095 | 2625 |
| 66 | 0.404857 | 0.413953 | 645 | 0.364423 | 0.363771 | 3373 |
| 67 | 0.35122 | 0.358811 | 471 | 0.393269 | 0.397295 | 1996 |
| 68 | 0.28539 | 0.302198 | 182 | 0.326412 | 0.330532 | 1071 |
| 69 | 0.221281 | 0.219373 | 351 | 0.359071 | 0.362224 | 2446 |
